# Overprescribing of potentially harmful medication: an observational study in England’s general practice

**DOI:** 10.3399/BJGPO.2023.0156

**Published:** 2024-06-12

**Authors:** Tasneem Khan, Bethan Copsey, Paul Carder, Stella Johnson, Mohammed Imran, Kaiwen Wang, Sarah Alderson

**Affiliations:** 1 Leeds Institute of Health Sciences, University of Leeds, Leeds, UK; 2 Clinical Trials Research Unit, University of Leeds, Leeds, UK; 3 NHS West Yorkshire Integrated Care Board, Leeds, UK; 4 University of Leeds, Leeds, UK

**Keywords:** analgesics, opioid, anti-inflammatory agents, non-steroidal, general practice, inappropriate prescribing, prescriptions, socioeconomic factors, United Kingdom, general practitioners, primary healthcare

## Abstract

**Background:**

Overprescribing of potentially harmful medication in UK general practice has a complex association with socioeconomic deprivation.

**Aim:**

To assess trends in general practice prescribing of five high-risk medications and their relationship with deprivation.

**Design & setting:**

An observational study was conducted using general practice data from three English regions with varied sociodemographic factors: West Yorkshire and Harrogate (WY), Black Country and West Birmingham (BC), and Surrey and East Sussex (SE).

**Method:**

Practice-level prescribing data were obtained from 2016–2021 for five drug classes: opioids, hypnotics, gabapentinoids, non-steroidal anti-inflammatory drugs (NSAIDs), and antibacterials. Prescribing trends were demonstrated using a linear model.

**Results:**

Reduction in NSAID, opioid, hypnotic and antibacterial prescriptions, and the increase in gabapentinoid prescriptions, were significant at each financial year time period. Index of Multiple Deprivation (IMD) was positively associated with all drug classes except antibacterials, which showed a positive association when incorporating the interaction term between IMD and age.

When adjusting for IMD and population, region was independently associated with prescribing rate. Compared with WY, IMD had a smaller association with prescribing in BC for NSAIDs (coefficient = −0.01578, *P* = 0.004) and antibacterials (coefficient = −0.02769, *P* = 0.007), whereas IMD had a greater association with prescribing in SE for NSAIDs (coefficient = 0.02443, *P*<0.001), opioids (coefficient = 0.08919, *P*<0.001), hypnotics (coefficient = 0.09038, *P*<0.001), gabapentinoids (coefficient = 0.1095, *P*<0.001), and antibacterials (coefficient = 0.01601, *P* = 0.19).

**Conclusion:**

The association of socioeconomic deprivation with overprescribing of high-risk medication in general practice varies by region and drug type. Geographical location is associated with overprescribing, independent of socioeconomic status.

## How this fits in

Potentially harmful and low-value medication is often overprescribed in UK general practice. Overprescribing is associated with socioeconomic deprivation, but the nature of the association is not clearly understood. We have shown that the effect of social deprivation on overprescribing differs by drug type and by region, and that geographical region is independently associated with rates of overprescribing. National prescribing targets must address regional challenges in implementation of guidelines. The role of inequity and intersectionality needs to be considered in health policy and implementation research.

## Introduction

Overprescribing of potentially harmful medication in UK general practice is associated with areas of socioeconomic deprivation.^1^ In 2018, the Department of Health and Social Care ordered a review into overprescribing, and subsequently introduced the NHS *Long Term Plan*, which included strategies to tackle overprescribing.^1,2^ The ‘Good for me, Good for you, Good for everyone’ review suggested that 10% of primary care medicine could be overprescribed.^3^ It also indicated that overprescribing was more prevalent in certain patient groups, including those living in areas of deprivation.^4^ Socially deprived regions tend to have an increased burden of chronic disease, which may contribute to overprescribing.^5^ Moreover, the mismatch between healthcare need and healthcare delivery runs deeper in deprived populations, potentially limiting access to alternative therapies.^6^ Variation by deprivation score has been demonstrated in primary care for opioid, benzodiazepine, and gabapentinoid prescribing.^7,8^ Antibiotic prescription and, therefore, antimicrobial resistance have also been linked to area-level deprivation.^9^

A measure of progress in the NHS *Long Term Plan* is reduction in unjustified performance variation. However, these inequalities cannot be addressed without identifying the nature of the variation. We hypothesise that although deprivation is a key determinant of prescribing variation, this alone does not explain the observed disparities. We undertook an observational study to assess trends in general practice overprescribing of high-risk medication and their association to deprivation. We also looked at whether the effect of deprivation on prescribing was uniform across different regions.

## Method

We chose five classes of drugs with high priority for deprescribing initiatives. Opioids, gabapentinoids, hypnotics, and antibiotics were selected due to their known association with area-level deprivation.^10^ Non-steroidal anti-inflammatory drugs (NSAIDs) were chosen, as there is increasing evidence of associated harm and the need for rationalised prescribing.^11^ Additionally, we chose lipid-modifying drugs as a comparator, where we would expect prescribing rates to rise with the greater push for cardiovascular risk assessment and the use of statin treatment in primary prevention.^12^

### Data collection

Routinely collected practice-level prescribing data from OpenPrescribing.com^13^ were acquired in monthly periods from January 2016 to March 2021. GP profiles were retrieved from the Fingertips data of the Office for Health Improvement and Disparities,^14^ which included yearly practice-level indicators for all practices in England, including the percentage of patients in each age category (0–4, 5–14, <18, >65, >75, >85 years), Index of Multiple Deprivation (IMD) scores and Quality and Outcomes Framework (QOF) score. QOF is an annual incentive programme for general practices, financially rewarding good clinical practice.^15^

### STP selection

In England, GPs work together in practices, with contracts of care relating to the practice rather than individual practitioners. Sustainability and transformation partnerships (STPs) were developed in 2016, covering populations of 1–3 million patients. They brought together NHS providers, Clinical Commissioning Groups (CCGs), and local authorities to plan services tailored to the local population. These were developed into integrated care systems (ICSs) in 2022, with the objective of tackling health inequalities.^16^ At the time of data collection, geographical regions were divided into STPs; therefore, we have continued to use this term for the purpose of this article. We chose three STPs with varied sociodemographic factors.

The West Yorkshire and Harrogate STP (WY), now West Yorkshire ICS, covers five CCGs: Bradford District and Craven, Calderdale, Leeds, Kirklees, and Wakefield. It supports 2.4 million people, with 35.1% of the population living in England’s most deprived quintile (as per population-level IMD score).^17^ Prescribing data were available for *n* = 324/326 practices.

Black Country and West Birmingham STP (BC), now Black Country ICS, merged its four CCGs into the single Black Country and West Birmingham CCG in April 2021. It supports 1.2 million people, with 45.7% of the population living in the most deprived quintile.^17,18^ Data were available for *n* = 3193/197 practices.

Sussex and East Surrey STP (SE), now Sussex ICS, covers three CCGs: Brighton and Hove, East Sussex, and West Sussex. It supports 1.7 million people, with 9.5% of the population living in the most deprived quintile.^18–20^ Data were available for all 246 practices.

BC is England’s second-most deprived STP. SE is among the least deprived, ranking 33rd out of 42. WY is ranked sixth least deprived, but has greater regional IMD score variation, ranging from areas ranking within England’s top 100 most deprived neighbourhoods (from a total 32 844), to those ranking among the 500 most affluent neighbourhoods.^18,21^

### Analysis

Data were collected for 763 practices and analysed to allow modelling of trends, as well as examining the effect of practice-level factors on high-risk prescribing. We investigated variability in prescribing over time, between practices, CCGs, and STPs, and between the different drug types at practice level.

We grouped the number and proportion of patients in each 5-year band age category, the number and proportion of patients by sex, and the total QOF points achieved by the mean, standard deviation, and range of total QOF points. Total QOF points were not available for 2020/21, as they were not reported due to the COVID-19 pandemic. IMD was available for 2015 and 2019, as population-level IMD scores are assessed every four years. We examined the mean, standard deviation, and range of IMD score for each CCG and STP.

The primary outcome was rate of prescribing (number of prescriptions divided by the number of registered patients) per 100 person-months for each time period. Practice-level data were aggregated at CCG and STP level. Multivariate subgroup analyses were conducted to explore differences associated with practice characteristics.

The trend in outcome over time was demonstrated using a linear model, including time as a dummy-coded categorical variable based on the QOF reporting period from 1 April to 31 March, using 2016/17 as the reference period. All regression models adjusted for the QOF period (categorical), IMD score (continuous), and total QOF score (continuous). For all drugs except antibiotics, we adjusted for the patient proportion aged >65 years. For antibacterial prescribing, we adjusted for patient proportion aged <18, since this was thought to be more clinically relevant; the proportion of patients aged <18 years was correlated with the patients aged >65 years.^22,23^

One regression model also adjusted for STP, including an interaction term between STP and IMD score. The reference category for STP was WY. A second regression model included an interaction term between IMD score and the age category for the corresponding drug, either the patient proportion aged <18 years or patient proportion aged >65 years .

For antibacterial prescribing, we excluded practices with <12 months of data, to prevent the effect of seasonality. For the remaining drugs, practices were included when ≥1 month of data were available; outcomes were converted to rate of prescribing for the corresponding number of person-months available. Data for the missing months were not imputed. Practices with missing covariates were excluded from the adjusted regression analyses.

To interpret the regression model and the magnitude of effect, we translated the coefficients into number of prescriptions. This was completed for the regression model with interaction term between IMD (2015) and STP. We calculated prescribing rates over 12 months for an arbitrary population of 2 million (as an approximation of an average STP). This translation assumed the same deprivation score between STPs, to negate the effect of IMD on prescription rates. Thus, we were able to deduce the effect of STP alone on variation in prescribing assuming the same population size and deprivation across the different regions. WY was used as the reference, as per the adjusted linear regression analysis.

## Results

The proportion of patients in each age category for all three STPs remained stable over time (Supplementary Tables 1 and 2). IMD score was similar in 2015 and 2019 for all STPs. Deprivation score was lower in SE than in WY and BC (Supplementary Table 3). We found a negative correlation between IMD 2015 and age >65 years in 2016 (correlation –0.68, *P*<0.0001, *n* = 678). These findings were similar between IMD 2019 and age >65 years in 2020 (correlation –0.69, *P*<0.0001, *n* =679) (Supplementary Figure 4). Seasonality was only seen with antibacterial prescribing; this effect was less pronounced in 2020/21 (Supplementary Figures 1–3).

### Descriptive statistics

**Figure 1.**
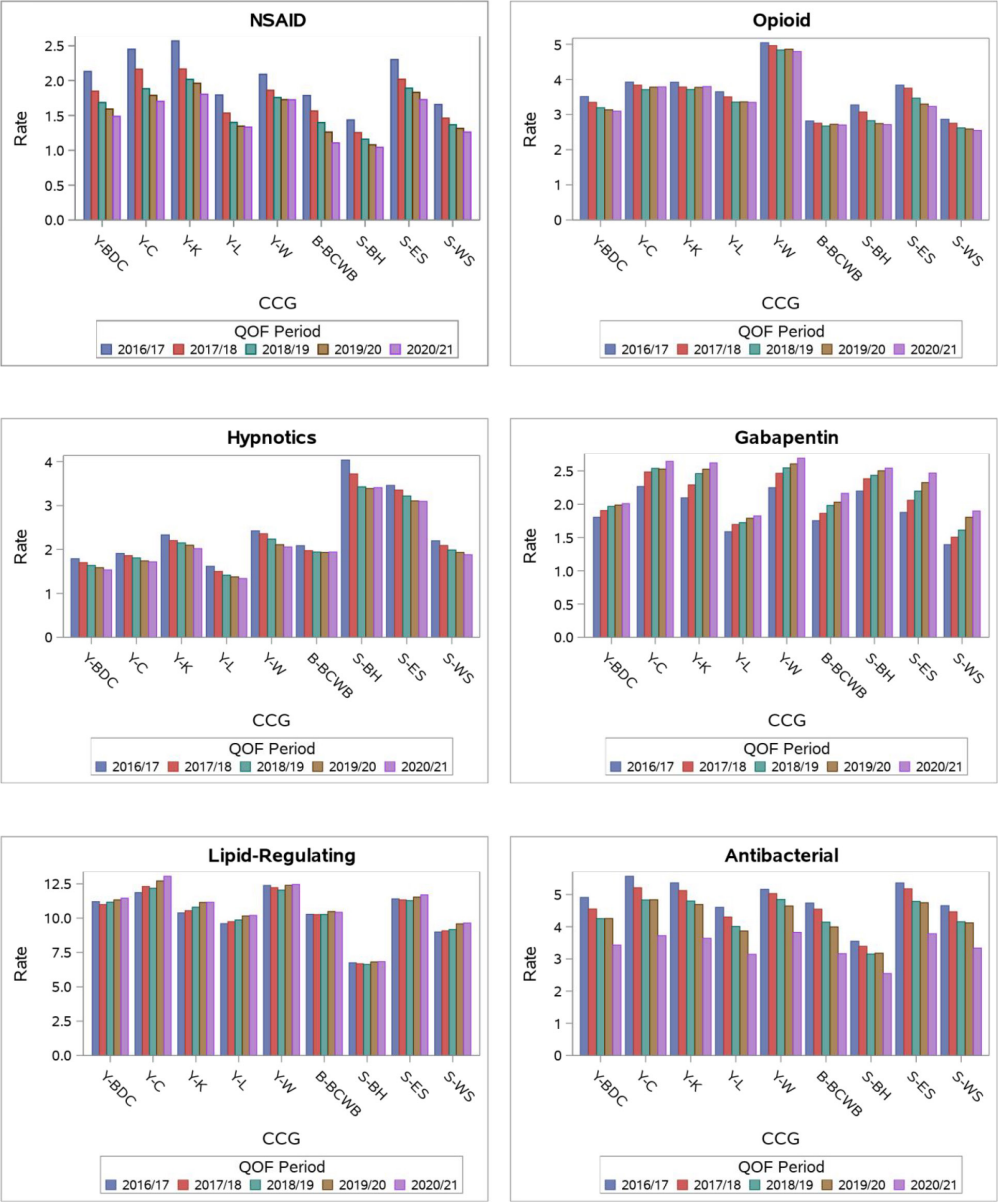
Prescribing rates per 100 person-months by CCG and time period. B-BCWB = Black Country and West Birmingham. NSAID = non-steroidal anti-inflammatory drug. QOF = Quality and Outcomes Framework. S-BH = Brighton and Hove. S-ES = East Sussex. S-WS = West Sussex. Y-BDC = Bradford District and Craven. Y-C = Calderdale. Y-K = Kirklees. Y-L = Leeds. Y-W = Wakefield. Accessibility note: QOF periods are shown from least to most recent (from 2016/17 to 2020/21) from left to right for each CCG.

Figure 1 shows the prescribing rates per 100 person-months by CCG and time period. The rate of NSAID prescriptions decreased in every STP, with overall decline from 1.94/100 person-months in 2016/17 to 1.47/100 person-months in 2019/20. The highest rate of NSAID prescription was found in WY for all time periods. The rate of opioid and hypnotics prescriptions also reduced over time, (3.42/100 person-months to 3.13/100 person-months, and 2.27/100 person-months to 1.99/100 person-months, respectively) with the greatest reduction seen in SE. Antibacterial prescriptions reduced over time in all STPs (4.83/100 person-months in 2016/17 to 3.34/100 person-months in 2020/21). Conversely, prescriptions for gabapentinoids increased in all areas (1.79/100 person-months in 2016/17 to 2.19/100 person-months in 2020/21). Our comparator, lipid-regulating prescriptions, saw a slight increase over time (10.20/100 person-months in 2016/17 to 10.59/100 person-months in 2020/21) (see Supplementary Tables 2.1–2.6).

### Regression analysis

Tables 1–5 show prescribing rates adjusted for baseline covariates. The reduction in prescriptions of NSAIDs, opioids, hypnotics, and antibacterials, and the increase in gabapentinoids, were significant at each QOF time period (*P*<0.0001). The change in rate of lipid-regulating drugs was significant in the time periods 2019/20 and 2020/21 (Supplementary Table 10). QOF score was only associated with antibacterial prescriptions (coefficient = 0.01667, *P* = 0.0489) (Table 5).

**Table 1.** Results of regression analyses on NSAIDs prescribing rate

			Interaction IMD x STP	Interaction IMD x age
	QOF period	STP	Coefficient, *P* value (95% CI)	Coefficient, *P* value (95% CI)
QOF period	17/18	_	–0.2673, 0.001	–0.2673, 0.0001
			(−0.2969 to −0.2377)	(−0.2969 to −0.2377)
	18/19	_	–0.4102, 0.001	–0.4102, 0.0001
			(−0.4399 to −0.3806)	(−0.4399 to −0.3806)
	19/20	_	–0.4859, 0.001	–0.4859, 0.0001
			(−0.5156 to −0.4563)	(−0.5156 to −0.4563)
	20/21	_	–0.5876, 0.001	–0.5876, 0.0001
			(−0.6173 to −0.5579)	(−0.6173 to −0.5579)
STP	_	BC	–0.3686, 0.001	_
			(−0.5041 to −0.2330)	
	_	SE	0.06911, 0.39	_
			(–0.08718, 0.2254)	
QOF score (2016/17)	_	_	0.00137, 0.76	0.000028, 0.99
			(–0.00743 to 0.01017)	(–0.00930 to 0.009357)
IMD score (2015)	_	_	0.02083, 0.001	0.01742, 0.0001
			(0.01441 to 0.02725)	(0.01176 to 0.02307)
Proportion >65 (2016)	_	_	0.03530, 0.001	0.03281, 0.0001
			(0.02571 to 0.04490)	(0.02249 to 0.04313)
Interaction: STP x IMD score (2015)^b^	_	BC	–0.01578, 0.004	_
			(−0.02661 to −0.00496)	
	_	SE	0.02443, 0.001	_
			(0.01238 to 0.03649)	
Interaction: proportion >65 (2016) x IMD score (2015)	_		_	0.000450, 0.15
				(–0.00017 to 0.001070)

BC = Black Country and West Birmingham. CI= confidence interval. IMD = Index of Multiple Deprivation. NSAID = non-steroidal anti-inflammatory drug. QOF = Quality and Outcomes Framework. SE = Surrey and East Sussex. STP = sustainability and transformation partnership.

**Table 2.** Results of regression analyses on opioids prescribing rate

			Interaction IMD x STP	Interaction IMD x age
	QOF period	STP	Coefficient, *P* value (95% CI)	Coefficient, *P* value (95% CI)
QOF period	17/18	_	–0.1081, 0.001	–0.1081, 0.001
			(−0.1491 to −0.06708)	(−0.1491 to −0.06713)
	18/19	_	–0.2409, 0.001	–0.2409, 0.001
			(−0.2818 to −0.1999)	(−0.2819 to −0.1999)
	19/20	_	–0.2443, 0.001	–0.2444, 0.001
			(−0.2853 to −0.2033)	(−0.2853 to −0.2034)
	20/21	_	–0.2656, 0.001	–0.2657, 0.001
			(−0.3067 to −0.2246)	(−0.3067 to −0.2246)
STP	_	BC	–1.1273, 0.001	_
			(−1.3835 to −0.8710)	
	_	SE	–0.04654, 0.76	_
			(–0.3420 to 0.2489)	
QOF score (2016/17)	_	_	0.001501, 0.86	0.002844, 0.75
			(–0.01513 to 0.01813)	(–0.01448 to 0.02017)
IMD score (2015)	_	_	0.03780, 0.001	0.05814, 0.001
			(0.02566 to 0.04993)	(0.04764 to 0.06864)
Proportion >65 (2016)	_	_	0.1238, 0.001	0.1288, 0.001
			(0.1057 to 0.1420)	(0.005012 to 0.007314)
Interaction: STP x IMD score (2015)	_	BC	–0.00226, 0.83	_
			(–0.02273 to 0.01821)	
	_	SE	0.08919, 0.001	_
			(0.06640 to 0.1120)	
Interaction: proportion >65 (2016) x IMD score (2015)	_	_	_	0.006163, 0.001
				(0.002048 to 0.004057)

BC = Black Country and West Birmingham. CI= confidence interval. IMD = Index of Multiple Deprivation. QOF = Quality and Outcomes Framework. SE = Surrey and East Sussex. STP = sustainability and transformation partnership.

**Table 3.** Results of regression analyses on hypnotics prescribing rate

			Interaction IMD x STP	Interaction IMD x age
	QOF period	STP	Coefficient, *P* value (95% CI)	Coefficient, *P* value (95% CI)
QOF period	17/18	_	–0.1081, 0.001	–0.1081, 0.001
			(−0.1407 to −0.07562)	(−0.1406 to −0.07557)
	18/19	_	–0.1911, 0.001	–0.1911, 0.001
			(−0.2237 to −0.1586)	(−0.2236 to −0.1586)
	19/20	_	–0.2396, 0.001	–0.2396, 0.001
			(−0.2721 to −0.2071)	(−0.2721 to −0.2071)
	20/21	_	–0.2630, 0.001	–0.2630, 0.001
			(−0.2956 to −0.2305)	(−0.2956 to −0.2305)
STP	_	BC	0.09554, 0.38	_
			(–0.1172 to 0.3083)	
	_	SE	1.6505, 0.001	_
			(1.4052 to 1.8958)	
QOF score (2016/17)	_	_	–0.0104, 0.14	–0.02080, 0.007
			(–0.02423 to 0.003380)	(−0.03591 to −0.00568)
IMD score (2015)	_	_	0.01032, 0.04	0.01887, 0.001
			(0.000251 to 0.02039)	(0.009709 to 0.02803)
Proportion >65 (2016)	_	_	0.05202, 0.001	0.06835, 0.001
			(0.03695 to 0.06708)	(0.05163 to 0.08507)
Interaction: STP x IMD score (2015)	_	BC	–0.00002, 0.99	_
			(–0.01702 to 0.01697)	
	_	SE	0.09038, 0.001	_
			(0.07146 to 0.1093)	
Interaction: proportion >65 (2016) x IMD score (2015)	_	_	_	0.003, 0.001
				(0.002048 to 0.004057)

BC = Black Country and West Birmingham. CI= confidence interval. IMD = Index of Multiple Deprivation. QOF = Quality and Outcomes Framework. SE = Surrey and East Sussex. STP = sustainability and transformation partnership.

**Table 4.** Results of regression analyses on gabapentinoids prescribing rate

			Interaction IMD x STP	Interaction IMD x age
	QOF period	STP	Coefficient, *P* value (95% CI)	Coefficient, *P* value (95% CI)
QOF period	17/18	_	0.1382, 0.001	0.1382, 0.001
			(0.09546 to 0.1809)	(0.09546 to 0.1809)
	18/19	_	0.2444, 0.001	0.2444, 0.001
			(0.2017 to 0.2872)	(0.2017 to 0.2872)
	19/20	_	0.3315, 0.001	0.3315, 0.001
			(0.2887 to 0.3742)	(0.2887 to 0.3742)
	20/21	_	0.4088, 0.001	0.4088, 0.001
			(0.3660 to 0.4516)	(0.3660 to 0.4516)
STP	_	BC	–0.1078, 0.41	_
			(–0.3647 to 0.1492)	
	_	SE	0.9920, 0.001	_
			(0.6958 to 1.2883)	
QOF score (2016/17)	_	_	0.002115, 0.80	–0.00488, 0.58
			(–0.01456 to 0.01879)	(–0.02237 to 0.01261)
IMD score (2015)	_	_	0.01599, 0.01	0.03397, 0.001
			(0.003825 to 0.02816)	(0.02336 to 0.04457)
Proportion >65 (2016)	_	_	0.04276, 0.001	0.05077, 0.001
			(0.02457 to 0.06096)	(0.03143 to 0.07012)
Interaction: STP x IMD score (2015)	_	BC	–0.00249, 0.81	_
			(–0.02301 to 0.01804)	
	_	SE	0.1095, 0.001	_
			(0.08660 to 0.1323)	
Interaction: proportion >65 (2016) x IMD score (2015)	_	_	_	0.0038, 0.001
				(0.001456 to 0.003780)

BC = Black Country and West Birmingham. CI= confidence interval. IMD = Index of Multiple Deprivation. QOF = Quality and Outcomes Framework. SE = Surrey and East Sussex. STP = sustainability and transformation partnership.

**Table 5.** Results of regression analyses on antibiotics prescribing rate

			Interaction IMD x STP	Interaction IMD x age
	QOF period	STP	Coefficient, *P* value (95% CI)	Coefficient, *P* value (95% CI)
QOF period	17/18	_	–0.2147, 0.001	–0.2147, 0.001
			(−0.2752 to −0.1541)	(−0.2752 to −0.1541)
	18/19	_	–0.5548, 0.001	–0.5548, 0.001
			(−0.6153 to −0.4943)	(−0.6154 to −0.4943)
	19/20	_	–0.6227, 0.001	–0.6226, 0.001
			(−0.6833 to −0.5622)	(−0.6832 to −0.5620)
	20/21	_	–1.4749, 0.001	–1.4748, 0.001
			(−1.5360 to −1.4137)	(−1.5360 to −1.4137)
STP	_	BC	–0.1786, 0.16	_
			(–0.4297 to 0.07248)	
	_	SE	–0.04664, 0.76	_
			(–0.3492 to 0.2559)	
QOF score (2016/17)	_	_	0.01667, 0.05	0.01563, 0.07
			(0.000079 to 0.03327)	(–0.00115 to 0.03241)
IMD score (2015)	_	_	–0.00798, 0.20	–0.01094, 0.02
			(–0.02026 to 0.004296)	(−0.01989 to −0.00199)
Proportion <18 (2016)	_	_	0.01183, 0.35	0.01425, 0.31
			(–0.01286 to 0.03653)	(–0.01327 to 0.04176)
Interaction: STP x IMD score (2015)^b^	_	BC	–0.02769, 0.007	_
			(−0.04786 to −0.00752)	
	_	SE	0.01601, 0.19	_
			(–0.00787 to 0.03989)	
Interaction: proportion <18 (2016) x IMD score (2015)	_	_	_	–0.00094, 0.27
				(–0.00261 to 0.000731)

BC = Black Country and West Birmingham. CI= confidence interval. IMD = Index of Multiple Deprivation. QOF = Quality and Outcomes Framework. SE = Surrey and East Sussex. STP = sustainability and transformation partnership.

The association of IMD score with prescribing rates was significant for all classes of drugs (*P*<0.05) except antibacterials (Tables 1 2 3 4). However, when incorporating the interaction term between IMD and age, the association for antibacterials became significant (coefficient = −0.01094, *P* = 0.0166) (Table 5).

The effect of IMD in each STP was investigated, compared with WY. IMD had a smaller association with prescribing rates in BC for NSAIDs (coefficient = −0.01578, *P* = 0.004) and antibacterials (coefficient = −0.02769, *P* = 0.007) (Tables 1 5). Conversely, IMD had a greater association with prescribing rates in SE for NSAIDs (coefficient = 0.02443, *P*<0.001), opioids (coefficient = 0.08919 *P*<0.001), hypnotics (coefficient = 0.09038, *P*<0.001), gabapentinoids (coefficient = 0.1095, *P*<0.001), and antibacterials (coefficient = 0.01601, *P* = 0.19) (Tables 1 2 3 4 5).

### Translation of regression coefficients

For NSAID prescriptions, the difference in rate between WY and BC was –0.3686 (per 100 person-months). This equates to 88 800 fewer NSAID prescriptions in BC per population of 2 million. Similarly, there were 16 800 more prescriptions in SE per population of 2 million. For opioids, there were 268 800 fewer prescriptions in BC, and 9600 fewer prescriptions in SE per population of 2 million. For hypnotics we found 23 040 fewer prescriptions in BC but 396 000 more prescriptions in SE per population of 2 million. For gabapentinoids, there were 25 920 fewer prescriptions in BC, but 238 080 more prescriptions in SE per population of 2 million. For antibacterials, there were 42 960 fewer prescriptions in BC, and 11 280 fewer prescriptions in SE per population of 2 million.

## Discussion

### Summary

Prescribing of opioids, NSAIDs, antibacterials, and hypnotics reduced over the period of January 2016 to March 2021, whereas gabapentinoid prescribing increased. Prescribing of high-risk medication had a significant association with IMD scores, the extent of which differed according to region. The effect of deprivation on prescribing of all five classes of drugs was greatest in SE.

STP alone had a significant association with prescribing rates, independent of IMD score. The extent of this association has been demonstrated through the translation of the regression coefficients, which shows the difference in number of prescriptions that can be attributed to STP alone.

We have shown that unwarranted clinical performance variation, as set out in the NHS *Long Term Plan* as a target measure, is only partly explained by area-level deprivation. This may be a result of local initiatives to tackle overprescribing, or a discrepancy in how national strategies are implemented according to geographical location. Differences in ethnicity and immigration could account for the STP effect, as could other practice-level factors, such as the degree of single-practitioner compared with multi-practitioner general practices.

### Strengths and limitations

Our study evaluated prescribing trends for five high-risk medications, in three locations, across a 5-year period; this allowed assessment of variability according to several indicators. The organisational structures are specific to England, but we believe our findings can be generalised to the UK. Although NHS health policy has devolved, the UK government retains power on many issues related to the health service.^24^

Our study has four limitations. First, the subgroup analysis showed a large variation in prescribing of hypnotics and antibiotics for those practices with missing data on covariates; however, this number is small, since those with 12 months of QOF data usually also provided Fingertips data. Overall, practices with missing covariate information had similar prescribing rates to those with covariate information.

Second, our analysis is limited by the potential of online databases to be incomplete. Patients who have moved between practices cannot be accounted for. Where practices have shut down, we assume that patients will remain registered within the locality. Our association analyses are limited by information available through Fingertips owing to the poor quality of reporting in health records. Thus, we were unable to assess other factors that may affect overprescribing, such as ethnicity.

Third, we selected regions from the north (WY), midlands (BC) and south of England (SE), but those areas not included may have different contexts to their IMD scores. There is existing evidence for health outcome discrepancy between the north and south of England,^25^ which may also limit the generalisability of our findings.

Finally, as our analysis took place during the period of STP transition to ICS, any discrepancy in geographical boundaries cannot be accounted for. For example, Harrogate CCG formed part of the West Yorkshire and Harrogate STP but moved into the Humber Coast and Vale ICS (accounting for *n* = 17/324 practices). Where CCGs have merged, the practices included in the dataset remain the same.

### Comparison with existing literature

Our findings confirm existing evidence for the relationship between overprescribing of high-risk medication and social deprivation;^3^ this is consistent across all five classes of drugs and all three regions. Research using UK general practice data across a 12-month period has shown that strength of association with deprivation is dependent on drug type;^26^ our study builds on these findings using longer-term data, and further demonstrates the effect of geographical location.

Social constructs influence health,^27^ but these are not limited to social class. The IMD score does not take into account ethnicity or migration, nor does it provide information regarding financial resources within a region.^28^ Our results indicate that IMD score alone does not explain the regional variation in overprescribing of potentially harmful medication in general practice.

### Implications for research and practice

Our study demonstrates that the association between general practice overprescribing and socioeconomic deprivation varies by region and by drug type, and geographical location is associated with overprescribing independent of socioeconomic status.

Regional health inequalities are widening in England.^25^ Overprescribing of high-risk medication contributes to morbidity and mortality, and measures to reduce variation in prescribing are essential to improving health outcomes.^3^ Prescribing targets are set nationally, but local factors influencing implementation need to be addressed. At policy level, decision-making needs to include marginalised groups^27^ and regional representations. Ongoing research into the drivers of health inequalities must acknowledge intersectionality. At practice level, health professionals have a role in addressing the social determinants of health,^29^ but support must be tailored to the local population.^17^ Quality improvement initiatives targeting overprescribing cannot employ a one-size-fits-all approach, and national programmes need to be adapted to local needs.

## Data

All data has been fully presented in this article.
